# Assessment of the Tolerance of Dobenox Forte^®^ in Patients with Chronic Venous Disease

**DOI:** 10.3390/life14040437

**Published:** 2024-03-25

**Authors:** Jerzy Chudek, Agnieszka Almgren-Rachtan, Agnieszka Pastuszka, Damian Ziaja

**Affiliations:** 1 Department of Internal Medicine and Oncological Chemotherapy, Medical Faculty in Katowice, Medical University of Silesia in Katowice, 40-027 Katowice, Poland; 2 Department of Pharmacovigilance, Europharma Research & Scientific Centre Co., Ltd., 40-061 Katowice, Poland; agnieszka@europharma.edu.pl; 3 Department of Descriptive and Topographic Anatomy, Faculty of Medical Science in Zabrze, Medical University of Silesia in Katowice, 41-808 Zabrze, Poland; 4 Department of Physiotherapy, School of Health Sciences in Katowice, Medical University of Silesia in Katowice, 40-752 Katowice, Poland; damian.ziaja@op.pl; 5 Department of Vascular Surgery at St. Barbara’s Hospital in Sosnowiec, 41-200 Sosnowiec, Poland

**Keywords:** calcium dobesilate, chronic venous disease, limb heaviness, calf cramps, calf circumference, lower legs swelling

## Abstract

This prospective, observational, multicenter study assessed the tolerance of Dobenox Forte^®^, the first approved over-the-counter product containing calcium dobesilate, in 1795 outpatients with chronic venous disease (CVD) in daily clinical practice. In addition, the effectiveness (decrease in circumferences of a more affected limb at the ankle and middle part of the calf, and changes in the severity of CVD signs) was assessed. No adverse events related to use of the preparation were reported in a period of 64 ± 20 days. Dobenox Forte^®^ use was associated with a reduction in calf circumference by 13.1 mm (95%CI: 12.2–14.1) and in ankle circumference by 9.7 mm (95%CI: 9.2–11.0) in patients reporting swelling of the lower legs (60.0% of the cohort). A reduction in calf and ankle circumference by at least 1 cm was achieved in 34.9% and 24.9% of patients, respectively. The percentages of patients reporting moderate to very severe lower limb heaviness decreased from 96.6% to 56.0%, calf cramps decreased from 91.0% to 41.0%, calf pain decreased from 89.2% to 43.7%, swelling decreased from 86.1% to 38.8%, and burning sensation that worsens when standing decreased from 79.0% to 33.7%. The medicinal product Dobenox Forte^®^ is well tolerated by patients and seems to effectively reduce the symptoms of CVD.

## 1. Introduction

Dobenox Forte^®^, the first over-the-counter generic medicinal product containing 500 mg of calcium dobesilate monohydrate in a tablet, was approved for marketing in 2014 as an active medicine with well-established use. The medicine has been used for more than 10 years and its efficacy and safety have been well established based on results from the scientific literature. A biocompatibility study was performed for Calcium Dobesilate Hasco^®^ (produced by the same company under another trade name with the same composition of the tablet) with the original preparation (Doxium^®^) [1].

Calcium dobesilate is a synthetic phlebotropic drug with antioxidant properties [2] that inhibits serotonin-, bradykinin, and histamine-induced capillary permeability [3], and the synthesis of prostaglandins and thromboxane A2, thereby reducing platelet and erythrocyte aggregation and lowering blood viscosity [4,5]. In humans, a significant decrease in the venous distensibility index, maximum venous outflow, and capillary filtration coefficient after a three-month treatment with calcium dobesilate was demonstrated with plethysmography in a randomized study compared to placebo [6]. The improvement of blood viscosity and coagulation parameters during the treatment with calcium dobesilate was shown in subjects with diabetic retinopathy [7], and the results were generalized to the other vascular beds. Similarly, the beneficial effects of calcium dobesilate on capillary fragility were demonstrated in patients with diabetic retinopathy [8] and then translated to changes in the microcirculation.

These vasoprotective actions were shown to contribute to a reduction in lymph production, and the prevention of peripheral edema formation [9], partially by decreasing albumin leakage [10]. In addition, patients with chronic venous disease (CVD) using calcium dobesilate, benefitted from decreased aggregation of platelets and increased elasticity of erythrocytes in the microcirculation [11]. Nevertheless, there is a lack of data showing a reduction in the incidence of thrombosis in CVD patients treated with calcium dobesilate.

The effectiveness of calcium dobesilate in the treatment of patients with CVD was summarized in a meta-analysis covering 10 randomized clinical trials [12]. Calcium dobesilate in a daily dose of 1.0 or 1.5 g reduced fluid retention in the lower limbs (volume) and alleviated CVD symptoms, reducing night cramps, discomfort, paresthesia, pain, and heaviness of the lower limbs. There was a larger improvement in ankle pain, paresthesia, heaviness, and swelling among patients with greater symptom severity. Since no significant benefits were found with the use of calcium dobesilate at a daily dose of 1.5 g, a dose of 1.0 g is considered optimal in the treatment of CVD.

Calcium dobesilate was placed among the phlebotropic drugs recommended by the European Society for Vascular Surgery (ESVS) [13]. These recommendations confirm the beneficial effect of calcium dobesilate on swelling and the feeling of pain, leg heaviness and cramps, itching, and paresthesia in symptomatic patients.

As no trial has been conducted to confirm the safety of Dobenox Forte^®^, the aim of this observational study was to assess the tolerance of this medicinal product in patients with CVD. In addition, the study analyzed the effectiveness of Dobenox Forte^®^ in daily clinical practice.

## 2. Materials and Methods

This prospective, observational, multicenter study involved 1795 patients using Dobenox Forte^®^ (tablets containing 500 mg of calcium dobesilate monohydrate in a tablet) for 14 to 30 days for treatment of CVD, prescribed according to the Summary Product Characteristics (SPC). The patients were managed by 211 general practitioners, surgeons, and angiologists (specialists or physicians under training). Only patients who agreed to participate (gave oral consent) were included. The short use of Dobenox Forte^®^ for the treatment of CVD was the only inclusion criterion. There were no exclusion criteria. The number of patients who refused to provide consent was not recorded. The study was implemented from July to December 2016.

The participation of patients in the study did not result in additional diagnostic procedures or additional monitoring. The assessment of Dobenox Forte^®^ tolerance and effectiveness was performed as part of two subsequent, routine, outpatient visits approximately 3 months apart. During the first visit, demographic and comorbidity data were collected. On the first and follow-up (second) visits, the severity of symptoms related to CVD was recorded, and circumferences of the more affected lower leg at the ankle and middle part of the calf were measured. All patients’ data were anonymized at the stage of completing the survey questionnaire by physicians.

### 2.1. Assessment of Dobenox Forte^®^ Tolerance

The analysis of tolerance (primary endpoint) included all patients who took at least one dose of Dobenox Forte^®^ [N = 1975]. All adverse drug reactions (ADRs) were recorded in the study questionnaire during the initial and follow-up visits.

### 2.2. Assessment of Dobenox Forte^®^ Effectiveness

The analysis of the medicinal product’s effectiveness (secondary endpoint) was performed based on measurements of the more affected limb circumferences at the ankle and middle part of the calf in a subset of patients previously not treated with calcium dobesilate-containing medications and not utilizing compression therapy. Circumference measurements were made with an accuracy of 5 mm. The reduction in the circumferences between the first and the follow-up visits was calculated during the analysis.

The assessment of the CVD symptoms’ severity (feeling of heaviness in the more affected limbs, pain in the calves, calf cramps, a burning sensation that increases when standing, swelling in the lower legs) was performed on a 4-point scale (mild, moderate, severe, very severe). This analysis was also restricted to those previously untreated with calcium dobesilate-containing medications and not utilizing compression therapy.

### 2.3. Data Analysis

The occurrence of ADRs reported during the initial and follow-up visits in the study cohort exposed to Dobenox Forte^®^ was the primary endpoint (tolerance).

The secondary study endpoints were the percentage of patients who experienced at least a 10 mm reduction in calf or ankle circumferences, and a reduction in the severity of symptoms associated with CVD. Patients who discontinued therapy or were previously treated with other calcium-dobesilate-containing medicine were excluded.

Nutritional status was defined according to WHO criteria. Being overweight was defined as a body mass index (BMI) of 25–29.9 kg/m^2^, while obesity as BMI > 30.0 kg/m^2^ [14]. Visceral obesity was scored based on the WC thresholds for Caucasians, according to the International Diabetes Federation (IDF): ≥94 cm in men and ≥80 cm in women [15].

### 2.4. Statistical Analysis

Digitization of anonymized questionnaires and data cross-checking were performed by the study organizer (Europharma Research & Scientific Centre Co., Ltd., Katowice, Poland). No data imputation was performed. All enrolled patients (N = 1795) with the confirmed, by physicians, use of Dobenox Forte^®^ were included in the safety analysis. In total, 90 patients’ records were excluded from the analysis of Dobenox Forte^®^’s efficacy, due to missing data on ankle or calf circumferences on enrolment (N = 67), the use of compression therapy (N = 9), or the previous use of other calcium-dobesilate-containing preparations (N = 14). In consequence, the analysis of Dobenox Forte^®^’s effectiveness included 1705 study participants.

Statistical analysis was performed using STATISTICA 10.0 PL software (Tibco Software Inc., Palo Alto, CA, USA). Qualitative data were presented as percentages and quantitative data as averages with standard deviations (SD) or 95% confidence intervals (95%CI). The distributions of qualitative variables were compared using the χ^2^ test. ‘*p*’ values < 0.05 were considered statistically significant.

## 3. Results

### 3.1. Study Group Characteristics

The study included the use of Dobenox Forte^®^ in 1795 patients, mostly women (72.9%) with CVD (Table 1). Concomitant diseases were reported in 58.3% of patients. The most common diseases in both study groups were arterial hypertension, coronary heart disease, arrhythmias, lipid disorders, and type 2 diabetes mellitus. There were no patients with a history of venous ulcers in the study group.

At the time of enrollment, Dobenox Forte^®^ was more frequently prescribed in the recommended dose of 500 mg twice a day—60.3%, while 39.7% were prescribed the dose once a day. Of note, only 0.5% of patients used compression therapy.

Before the prescription of Dobenox Forte^®^, 26.2% of patients were treated pharmacologically. Previously used phlebotrophics, most often containing diosmin, are listed in Table 1. Of those, calcium dobesilate preparations were previously used by 14 patients (0.8%). In addition, nine patients (0.5%) were utilizing compression therapy. These subsets of patients were excluded from the analysis of Dobenox Forte^®^’s effectiveness.

### 3.2. Dobenox Forte^®^ Tolerance

No adverse events related to use of the preparation were reported in the study cohort (N = 1795). There was no case of discontinuation of the therapy due to a lack of tolerance to the evaluated medicinal product.

### 3.3. Dobenox Forte^®^‘s Effectiveness

During the period of observation covering 64 ± 20 days of Dobenox Forte^®^ use, between the first and second visit, 32 (1.6%) patients discontinued therapy due to the disappearance of, or reduction in, symptoms (N = 13), the cost of the therapy (N = 16), the development of a thrombosis episode (N = 1), and the recommendations of another specialist (N = 2).

In the analyzed cohort (N = 1705), 60.0% of the analyzed cohort reported swelling of the lower legs. After 65 ± 19 days of Dobenox Forte^®^ use, in this subgroup, the calf circumference of more affected limbs decreased by 13.1 ± 1.2 (95%CI: 12.2–14.1) mm and the ankle circumference decreased by 9.7 ± 10.7 (95%CI: 9.2–11.0) mm (Table 2).

A reduction in calf or ankle circumferences by at least 1 cm was achieved in 34.9% and 24.9% of patients. The effectiveness was not related to the prescribed daily dose of Dobenox Forte^®^ (Table 2).

**Figure 1:**
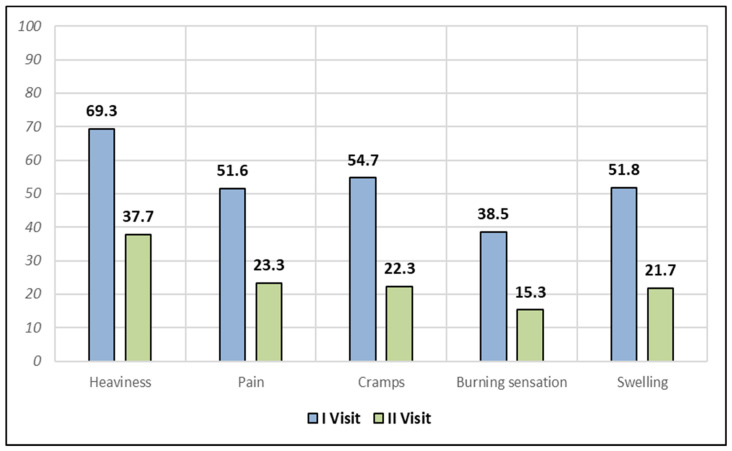
Percentages of patients reporting moderate to very severe symptoms of chronic venous disease during two subsequent visits (N = 1705).

The severity of reported symptoms significantly decreased (Table 3, Figure 1). The percentages of patients with moderate to very severe lower-limb heaviness decreased from 69.3% to 37.7%, calf cramps decreased from 54.7% to 22.3%, calf pain decreased from 51.6% to 23.3%, swelling decreased from 51.8% to 21.7%, and the burning sensation that worsens when standing decreased from 38.5% to 15.3%. There was no effect on the daily dose of Dobenox Forte^®^ on the achieved reduction of symptoms (Table 3).

## 4. Discussion

The results of the presented study confirm the safety and effectiveness of Dobenox Forte^®^ in the treatment of CVD in real-life settings.

Our study did not add much regarding the safety. During the period of observation covering 64 ± 20 days of Dobenox Forte^®^ use, no ADR was reported, confirming that this drug is one of the best-tolerated phlebotropic drugs, rarely causing gastrointestinal symptoms [16]. It must be mentioned that in 1992 calcium dobesilate was accused of causing agranulocytosis based on a single case of recurrent agranulocytosis after re-exposure to the drug [17]. Of note, the analysis of all reported cases of agranulocytosis in the years 1978–2000 identified only nine cases in which the relationship with calcium dobesilate use was highly probable [18]. Therefore, this risk of agranulocytosis is very small and does not justify monitoring of total blood counts during its use. In addition, a case of calcium dobesilate-induced hyperpyrexia was recently reported [19]. This association remains uncertain due to the lack of re-exposition to the drug after obtaining the remission of signs and symptoms.

In the assessment of the effectiveness of the treatment of CVD, the reduction in the calf circumference of the limb affected by the disease is more reliable than the subjective reduction of symptoms. In the analyzed subgroup reporting swelling, after 65 ± 19 days of Dobenox Forte^®^ use, the calf circumference of more affected limbs decreased by 13.1 (95%CI: 12.2–14.1) mm, and the ankle circumference decreased by 9.7 (95%CI: 9.2–11.0) mm. As much as one-third (34.9%) of these patients obtained at least a 1 cm reduction in calf circumference. It should be emphasized that the improvement was achieved exclusively without compression therapy, which was used by only 0.5% of the study patients, and excluded from the analysis of the efficacy. However, we cannot exclude that changes in the management of other than CVD causes of peripheral edema participated in the reduction of lower leg swelling.

In the largest previously conducted studies, the reduction in calf circumference was on average 3.3 mm after 3 months of using calcium dobesilate at a dose of 500 mg twice daily [20] and after 8 weeks of using the originator (Doxium^@^) at a dose of 500 mg three times a day [21]. The average reduction in the volume of the affected limb, after 8 weeks of treatment with calcium dobesilate in patients not using compression therapy was 1.82%, while 2.59% when combined with compression therapy [20]. This study confirmed the greater effectiveness of the use of calcium dobesilate in combination with compression therapy. Of note, the reduction in limb volume observed in this study (by 65 mL) was similar to that obtained with the solitary use of second-degree compression therapy, estimated at 34.6–54.4 mL. Importantly, when calcium dobesilate was used at the full recommended dose, limb volume reduction was achieved in 88% of patients [9].

When analyzing the effectiveness of the use of calcium dobesilate, the results of a meta-analysis by Martinez-Zapata et al. have to be mentioned [22]. This analysis showed a higher effectiveness of calcium dobesilate compared to placebo—a greater reduction in calf circumference by 1.7 (−1.5–4.8) mm. The effectiveness of calcium dobesilate was only inferior to that for diosmin preparations—pointing reduction of 6.0 (95%CI: 4.2–7.8) mm. Contrary to these data, our study shows absolute, but not relative to placebo changes in calf circumferences in a subset of patients reporting leg swelling. The reported absolute values are always greater than the relative ones against the placebo.

As previously shown limb heaviness (72.9%), ankle swelling (68.4%), and nighttime leg cramps (58.6%) are the most frequent CVD signs reported by patients, with a difficult-to-explain significant territorial diversity in the prevalence at least across Poland [23]. It seems that the prevalence of symptoms could be affected by the judgment of the cause of symptoms in patients with multimorbidity, which may be dependent on some regional differences in pre- and postgraduate training of physicians. From a global perspective, the prevalence of varicose veins was shown to be highest in Western Europe and lowest in the Middle East and Africa [24]. Of note, a large part of the participants of this study obtained improvement in CVD signs. There was a significant relief of signs of cramps, burning sensation, pain, swelling, and limb heaviness. These data are in line with ESVS guidelines concerning the effectiveness of calcium dobesilate in CVD [13].

It is worth noting that Dobenox Forte^®^ was similarly effective when used once and twice a day. However, interpretation in this aspect must be very careful due to the observational nature of the study, and the lack of knowledge concerning causes of differences in the prescribed doses of calcium dobesilate. This observation may also suggest the coexistence of the overlap with other diseases manifested by edema (heart failure, chronic kidney disease) and ADRs related to the use of calcium channel blockers in the therapy of hypertension [25,26]. Arterial hypertension was the most frequent comorbidity in the study cohort (48.4%). However, we did not collect data concerning the comedication of other diseases than CVD, calcium channel blockers are frequently used in hypertensive patients. A recent large nationwide Polish cohort study, performed among 12,289 patients with hypertension showed that as much as 45.7% of them were prescribed one of calcium channel blockers containing medicines [27].

The limitations of this large study are related to its methodology. It was an open-label cohort study performed in daily clinical practice settings. The effectiveness of the Dobenox Forte^®^ therapy was not placebo-controlled, and it was assessed in patients already, however shortly (up to 30 days), treated with Dobenox Forte^®^. As a study performed in daily clinical practice, it enrolled a significant percentage of patients with coexisting comorbidities (58.3%). Of note, the modification therapy of comorbidities could generate some bias related to the management of edema related to other than CVD clinical conditions (e.g., heart failure, chronic kidney disease). On the other hand, these comorbidities may explain the choice of calcium dobesilate-containing medicine by physicians in the management of CVD. Due to the pharmacodynamic properties of calcium dobesilate, namely poorly binding to proteins and no influence on the hepatic metabolism of other drugs [28]. Therefore, Dobenox Forte^®^ could be safely used along with numerous polytherapies, with a high compliance rate typical for phebotophics [29].

Having in mind the limited accuracy of the performed measurements we took stringent criteria for the primary endpoint—reduction of calf or ankle circumferences of at least 10 mm and restricted the analysis of effectiveness to patients reporting swelling of the lower legs.

## 5. Conclusions

This observational study demonstrates that the medicinal product Dobenox Forte^®^ is well tolerated by patients and seems to effectively reduce the symptoms of CVD.

## Figures and Tables

**Table 1:** Characteristics of the whole group (N = 1795) and patients included in the analysis of Dobenox Forte^®^’s effectiveness (N = 1705).

	Whole Group (N = 1795)	Included in the Analysis of Efficacy (N = 1705)
Age (years)	57.4 ± 14.0	58.2 ± 13.9
Women (N; %)	1309; 72.9	1240; 72.7
Nutritional status:		
Overweight—BMI 25–29.9 kg/m^2^ (N; %)	849; 47.3	803; 47.1
Obesity—BMI ≥ 30 kg/m^2^ (N; %)	533; 29.7	501; 29.4
Visceral obesity:		
Women (waist circumference ≥ 80 cm) (N; %)	1300; 72.4	1240; 72.7
Men (waist circumference ≥ 94 cm) (N; %)	862; 48.0	826; 48.5
CVD symptoms		
Varicose veins of the lower limbs (N; %)	984; 55.1	929; 54.5
Heaviness (N; %)	1287; 71.7	1273; 74.7
Pain (N; %)	1023; 57.0	1010; 59.2
Cramps (N; %)	1063; 59.2	1028; 60.3
Burning sensation (N; %)	874; 48.7	825; 48.4
Swelling (N; %)	1023; 60.0	1013; 59.4
Duration of CVD:		
<1 year (N; %)	294; 16.4	271; 15.9
1–5 years (N; %)	880; 49.0	839; 49.2
>5 years (N; %)	621; 34.6	595; 34.9
Co-morbidity (N; %)	1046; 58.3	988; 57.9
Hypertension (N; %)	878; 48.9	825; 48.4
Coronary heart disease (N; %)	232; 12.9	216; 12.7
Past myocardial infarction (N; %)	36; 2.0	34; 2.0
Heart arrhythmias (N; %)	120; 6.7	113; 6.6
Heart failure (N; %)	77; 4.3	76; 4.5
Stroke (N; %)	29; 1.6	24; 1.4
Chronic kidney disease (N; %)	23; 1.3	19; 1.1
Diabetes type 1 (N; %)	9; 0.5	9; 0.5
Diabetes type 2 (N; %)	113; 6.3	113; 6.6
Asthma/COPD (N; %)	50; 2.8	48; 2.8
Dyslipidemia (N; %)	133; 7.4	132; 7.7
Hypothyroidism (N; %)	41; 2.3	41; 2.4
Degenerative changes in the spine (N; %)	47; 2.6	43; 2.5
The prescribed dose of Dobenox Forte^®^:		
500 mg once a day (N; %)	713; 39.7	688; 40.4
500 mg twice a day (N; %)	1082; 60.3	1017; 59.6
Compression therapy (N; %)	9; 0.5	0
Phlebothrophics used before Dobenox Forte^®^ (N; %)	470; 26.2	451; 26.4
* Ruscus aculeatus* extracts (N; %)	61; 3.4	60; 3.5
Diosmin (N; %)	359; 20.0	345; 20.2
Horse chestnut extracts (N; %)	14; 0.8	13; 0.8
Calcium dobesilate (N; %)	14; 0.8	0
Sulodexid [N, %]	7; 0.4	7; 0.4

Abbreviations: BMI—body mass index, COPD—chronic pulmonary disease.

**Table 2:** Changes in calf and ankle circumferences in patients diagnosed with chronic venous disease (N = 1705).

	I Visit	II Visit
Whole group	N = 1705	N = 1673
Patients reporting swelling of the lower legs (N; %)	1023; 60.0	1008; 60.3
Ankle circumference (cm)	25.2 ± 5.7	24.4 ± 5.6 **
Δ of ankle circumference (mm)	-	−9.7 ± 10.7
Patients with reduced ankle circumference ≥ 10 mm (N; %)	-	253; 24.9
Missing data (N)	47	49
Calf circumference (cm)	38.8 ± 7.8	37.6 ± 7.6 **
Δ of calf circumference (mm)	-	−13.1 ± 11.6
Patients with reduced calf circumference ≥ 10 mm (N; %)	-	352; 34.9
Missing data (N)	47	44
**500 mg once daily**	N = 677	N = 664
Patients reporting swelling of the lower legs (N; %)	390; 57.7	385; 57.9
Ankle circumference (cm)	26.2 ± 4.9	25.5 ± 5.5 ^
Δ of ankle circumference (cm)	-	−9.3 ± 10.1
Patients with reduced ankle circumference ≥ 10 mm (N; %)	-	86; 22.2
Missing data (N)	10	20
Calf circumference (cm)	39.1 ± 5.9	37.9 ± 6.6 *
Δ of calf circumference (mm)	-	−13.2 ± 10.5
Patients with reduced calf circumference ≥ 10 mm (N; %)	-	133; 34.5
Missing data (N)	11	12
**500 mg twice daily**	N = 1028	N = 1009
Patients reporting swelling of the lower legs (N; %)	633; 61.5	623; 61.7
Ankle circumference (cm)	24.5 ± 6.1	23.6 ± 5.5 *
Δ of ankle circumference (mm)	-	−10.0 ± 11.2
Patients with reduced ankle circumference ≥ 10 mm (N; %)	-	167; 26.8
Missing data (N)	37	29
Calf circumference (cm)	38.6 ± 8.9	37.4 ± 8.2 *
Δ of calf circumference (mm)	-	−13.1 ± 12.3
Patients with reduced calf circumference ≥ 10 mm (N; %)	-	219; 35.2
Missing data (N)	36	32

Statistical significance: ^ *p* < 0.1; * *p* < 0.05; ** *p* < 0.01.

**Table 3:** Assessment of the clinical symptoms of chronic venous disease during two subsequent visits (N = 1705).

	I Visit					II Visit					χ^2^ for Trend *p*
	*No Signs*	*Mild*	*Moderate*	*Severe*	*Very Severe*	*No Signs*	*Mild*	*Moderate*	*Severe*	*Very Severe*	
**Whole group (N = 1705)**											
Heaviness	28.3	2.4	16.0	38.6	14.7	32.5	29.8	32.1	5.5	0.1	<0.001
Pain	42.2	6.2	17.5	26.5	7.6	46.7	30.0	19.6	3.6	0.1	<0.001
Cramps	39.9	5.4	19.3	26.3	9.1	45.6	32.1	19.8	2.3	0.2	<0.001
Burning sensation	51.3	10.2	19.3	13.7	5.5	54.6	30.1	13.3	1.7	0.3	<0.001
Swelling	39.9	8.3	23.2	21.3	7.3	44.0	34.3	17.9	3.5	0.3	<0.001
**500 mg once daily (N = 677)**											
Heaviness	23.6	1.9	19.6	37.5	17.4	28.7	31.7	32.8	6.8	0.0	<0.001
Pain	37.3	6.8	19.8	26.6	9.5	40.9	37.4	17.6	4.1	0.0	<0.001
Cramps	37.2	4.1	18.8	29.6	10.3	40.0	33.6	22.8	3.3	0.3	<0.001
Burning sensation	47.0	10.1	21.7	13.6	7.6	47.9	34.7	16.0	1.4	0.0	<0.001
Swelling	37.7	8.7	26.4	19.6	7.6	40.4	38.2	18.4	3.0	0.0	<0.001
**500 mg twice daily (N = 1028)**											
Heaviness	31.1	2.7	13.9	39.2	13.1	34.8	28.6	31.7	4.7	0.2	<0.001
Pain	45.1	5.9	16.1	26.4	6.5	50.2	25.5	20.8	3.3	0.2	<0.001
Cramps	41.4	6.2	19.6	24.4	8.4	48.8	31.2	18.0	1.8	0.2	<0.001
Burning sensation	53.7	10.3	17.9	13.8	4.3	58.6	27.3	11.6	2.0	0.5	<0.001
Swelling	41.1	8.1	21.4	22.3	7.1	46.2	31.9	17.6	3.8	0.5	<0.001

## Data Availability

The datasets are available from the Europharma Research & Science Centre Co., Ltd. on a reasonable request (agnieszka@europharma.edu.pl).
